# Screening and Interventions for Cardiovascular Disease Prevention in the Limpopo Province, South Africa: Use of the Community Action Model

**DOI:** 10.3390/metabo12111067

**Published:** 2022-11-04

**Authors:** Peter M. Mphekgwana, Kotsedi D. Monyeki, Tebogo M. Mothiba, Mpsanyana Makgahlela, Nancy Kgatla, Rambelani N. Malema, Tholene Sodi

**Affiliations:** 1 Research Administration and Development, University of Limpopo, Polokwane 0700, South Africa; 2 Department of Physiology and Environmental Health, University of Limpopo, Polokwane 0700, South Africa; 3 Faculty of Health Science, University of Limpopo, Polokwane 0700, South Africa; 4 Department of Psychology, University of Limpopo, Polokwane 0700, South Africa; 5 Department of Nursing Science, University of Limpopo, Polokwane 0700, South Africa

**Keywords:** non-communicable diseases, cardiovascular diseases, CAM, metabolic syndrome

## Abstract

The rise in non-communicable diseases (NCDs) has been attributed to economic growth in developing countries, shifts in societal norms, and behaviors such as dietary habits and physical activity. Up to 80% of NCDs could be prevented by eliminating shared risk factors, mainly tobacco use, unhealthy diets, physical inactivity, and the harmful use of alcohol. The South African government’s national strategic plan to control NCDs, which includes cardiovascular disease (CVD) prevention, places a strong emphasis on the need to improve the prevention, detection, early intervention, and management of NCDs. In line with the above recommendations, this study aimed to screen rural communities using the non-laboratory INTERHEART Risk Score tool (NLIRS) and develop relevant and suitable intervention strategies for a patient at moderate risk of developing a heart attack. A quantitative research approach applying a household-based design was used to conduct this study and the community action model (CAM). The difference between pre-intervention and post-intervention results were analyzed using a *t*-test and Analysis of covariance (ANCOVA) with age, smoke, hypertension, and diabetes as the covariates. The study found a significant difference in proportions between pre and post-intervention for raised Systole (SBP), obesity by body mass index (BMI), and waist circumference (WC). In rural communities, using CAM to improve knowledge and behavioral practices of NCD risk factors is feasible and effective. This basket of interventions will assist community members in reducing their risk of developing metabolic syndromes as well as their risk of developing CVDs. Continued investment and research in CVD prevention interventions are required to improve health, reduce costs, and have long-term benefits for conflict-affected individuals and communities.

## 1. Introduction

Despite the advancements made in the healthcare system to combat the epidemic of non-communicable diseases (NCDs), conditions such as cancer, cardiovascular diseases (CVDs), and chronic obstructive pulmonary diseases (COPDs) have continued to account for a considerable proportion of the disease burden in developing countries [1]. Over 15 million people per year die due to an NCD; 85% of these preventable deaths take place in low- and middle-income countries [1]. Several risk factors, including genetic, metabolic, behavioral, and environmental factors both modifiable and non-modifiable, contribute to the development of NCDs [1]. There is no reason to doubt that unplanned or unsustainable urbanization and lifestyle changes will have a negative impact on health in Sub-Saharan Africa (SSA) as well as other regions [2,3]. If urgent action is not taken, the morbidity and mortality from NCDs in SSA are predicted to overtake those from infectious diseases by the year 2030 [4].

A study by Olmen and colleagues distilled three major issues for the management of chronic diseases in SSA as: rapid scale-up approaches through the public health approach; community and peer support; and strengthening of the health services in which care is embedded [5]. Health promotion, a change in lifestyle, and nurse training in CVD management are among the CVD prevention strategies that are recommended [6,7,8]. Additionally, prior studies have suggested that identifying communities’ epidemiological profiles in terms of their level of CVD risk, followed by the appropriate interventions, may help to lower the prevalence of CVD [9,10]. Based on these suggestions, the current study’s objectives were to screen rural communities using the non-laboratory INTERHEART Risk Score tool, implement a comprehensive CVD prevention program, and train community health workers (CHWs) to conduct health education, risk assessments, and motivational interviewing and promote cardiovascular health and education. This study is part of a larger project known as “SPICES”. SPICES is an acronym for Scaling up Packages of Interventions for Cardiovascular disease prevention in selected sites in Europe and sub-Saharan Africa. The partners implementing SPICES and their respective countries are: the University of Antwerp, Belgium (Coordinator); Brighton and Sussex Medical School and Nottingham Trent University, both in the United Kingdom; Brest University in France; and the University of Limpopo in South Africa.

The majority of deaths globally each year are caused by NCDs, with CVDs being the major contributor to the global burden of disease [11,12]. By 2030, it is anticipated that 24 million people will die globally from CVDs of which 75% live in low- and middle-income countries [13,14], with hypertension and diabetes being the major contributors to the global burden of disease [14,15]. Despite the improvement in the healthcare system, CVDs continue to take up a large share of the disease burden in South Africa. According to reports, CVD is the second leading cause of death after HIV/AIDS, killing approximately 210 people daily [16,17]. If urgent action is not taken, it is anticipated that the rate of CVD-related premature deaths among South African adults will rise by 41% by 2030 [18]. 

It is of concern that people who die from CVD in low- and middle-income settings often do not have the benefit of integrated primary healthcare (PHC) programs for prevention and early detection (cite source). There is a lack of awareness regarding the risk factors associated with CVDs and treatments as compared to those in high-income settings [19,20]. As a result, some individuals in low- and middle-income countries frequently experience a late diagnosis and succumb to CVDs and other NCDs during their prime years of productivity. The exorbitant costs of NCDs, including treatment, which is often lengthy and expensive, combined with the loss of income, force millions of low- and middle-income people into poverty annually and stifle development [1].

The World Health Organisation (2021) reported that concentrating on lowering the risk factors associated with these diseases is a crucial step in the control of NCDs [1]. Governments and other stakeholders have access to low-cost solutions to lessen the prevalent modifiable risk factors. To set policies and priorities, it is crucial to monitor the development and trends of NCDs and their risk [1,6,7,21,22]. The South African government’s national strategic plan to control the NCDs, which includes CVD prevention, places a strong emphasis on the need to improve the prevention, detection, early intervention, and management of NCDs [23]. However, previous studies conducted in SSA have identified several facilitators and barriers related to the prevention of CVDs, which include structural, organizational, professional, and patient-related factors and attitudinal components [24]. In the South African context, a study conducted in the same study setting identified the five key facility barriers as poor infrastructural development; shortage of medical supplies and equipment; lack of health promotion activities; shortage of nurses and other healthcare personnel; and poor accessibility to primary healthcare services [25]. In view of these few studies, which were conducted in South Africa and aimed to identify communities’ epidemiological profiles in terms of their level of CVD risk [16,17,18], it is unfortunate that these findings were not followed by suitable interventions. Therefore, the current study aims to assess the CVD risk in rural communities and create an all-encompassing CVD prevention program.

## 2. Materials and Methods

### 2.1. Study Setting and Design

This was an implementation research study aiming to scale-up packages of interventions for CVD in primary healthcare facilities in the Limpopo Province. Health promotion/education, CVD risk profiling, communication, care and treatment, and self-management with follow-up were the targeted interventions implemented for improved CVD prevention and care. The study was conducted in the Capricorn district in Limpopo Province namely, the Ga-Molepo villages (located in the eastern part of Polokwane city). The rural villages are serviced by 6 public Primary Health Care (PHC) clinics. The site was selected based on the findings and recommendations of research studies that reported a high prevalence of chronic diseases and an increase in the risk factors associated with the development of CVDs [26,27]. Health interventions to control CVD risk factors were also recommended in these previous studies. A quantitative research approach applying a descriptive cohort study was used to conduct this study. Participants were sampled based on exposure, and the outcome was assessed during follow-up. Follow-up data were collected in the households of the participants using the non-laboratory INTERHEART Risk Score tool.

### 2.2. Population and Sampling

**Figure 1:**
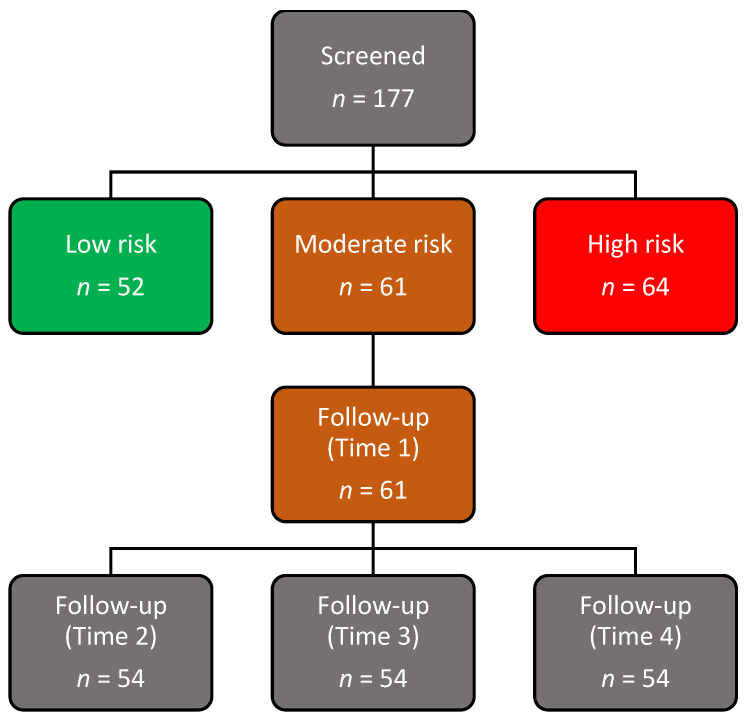
Schematic flowchart of the community cohort study.

The study idea was to profile participants for CVD risk in respective study areas before the intervention. For profiling and interventions, services of Community Health Workers (CHWs) linked to identifying healthcare facilities were trained on CVD knowledge, profiling, health promotion, and self-management. In each facility, designated professional nurses supervised CHWs at every phase of the project. In some instances, retraining in some aspects was necessitated and offered. Assuming confidence of 95%, a margin of error of 10%, and a conservative prevalence estimate of 33% [26] for the moderate risk group, the initial sample size was set at 85 for the intervention study. CVD risk profiling was conducted on 177 participants. Of these, more than a quarter (35%) of the participants were at a moderate risk, 36% high, and 29% at a low risk of developing CVD. The intervention was thus only implemented in the moderate group in the study shown in Figure 1.

### 2.3. Ethical Considerations

In preparation for data collection, ethical clearance was obtained from the University of Limpopo Turfloop Research and Ethics Committee (TREC/381/2017). Permission to conduct the study was obtained from the Limpopo Department of Health Research Committee. All the screened participants were briefed by trained CHWs about the data collection procedure before consenting to participate. Participants were further informed that their participation in the study was voluntary and that they were free to withdraw from the study at any time. A consent form was signed by those who agreed to participate in the study. Anonymity was ensured and all data was kept secure.

### 2.4. Community Action Model (CAM)

**Figure 2:**
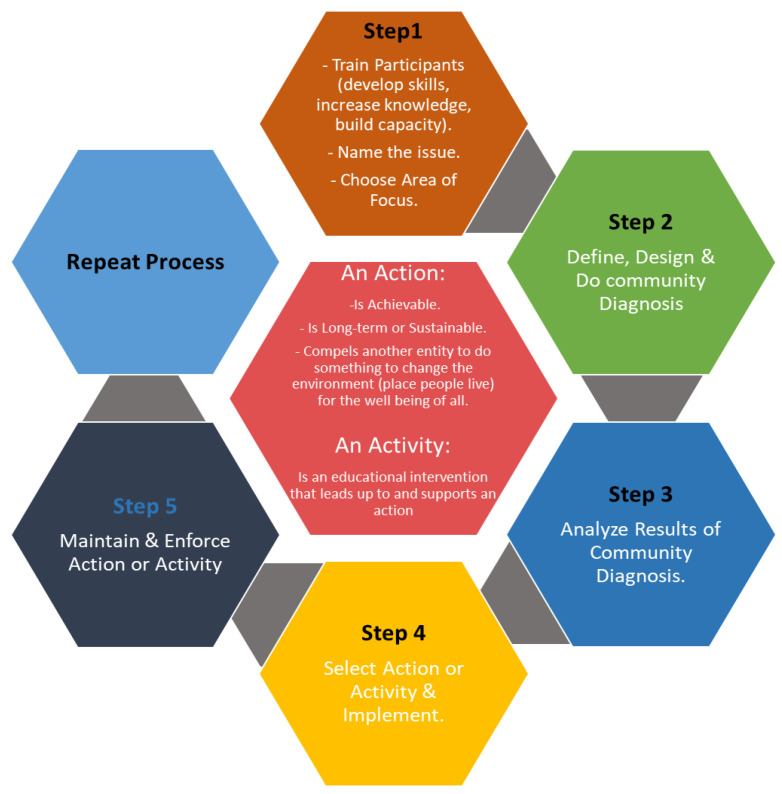
Community action model process (Adapted from “Community Action Model: Creating Change by Building Community Capacity,” by San Francisco Tobacco-Free, 2016, https://sanfranciscotobaccofreeproject.org/actions/community-action-model/ (accessed on 1 June 2022)).

Step 1: Train. The study partnered with five community-based clinics to recruit and build the capacity of CHWs to screen for CVD risk factors and counseling on adherence to lifestyle interventions and treatment among rural communities. The CHWs received training on how to conduct health education, risk assessments, and motivational interviewing, and promote cardiovascular health and education. Training with CHWs assessed baseline knowledge and skills and informed the development of the training curriculum and technical assistance provided to the cohort, implementing an enhanced CVD prevention program.
Step 2: Diagnosis. CHWs carried out community screening and profiling for the risk of developing a CVD during the second stage. In the diagnosis stage, community members aged 18 were screened utilizing quantitative research techniques and the non-laboratory INTERHEART Risk Score instrument.
Step 3: Analysis. The third step entailed analyzing the results of the profiled communities to identify community members at a moderate and high risk of developing a CVD.
Step 4: Implementation. This phase entailed the implementation of comprehensive CVD preventive measures, such as self-management strategies through educational programs that encouraged patients to engage in more physical activity and eat healthily for those who were at a moderate risk. Those who were found to be at a high risk were referred to the local clinics with a letter of recommendation from the CHW.
Step 5: Enforce. The action’s enforcement is the last stage to guarantee that the intended CVD screening and prevention continue in rural communities. CHWs serve as the foot soldiers throughout each stage, while government entity officials serve as the coaches. A CVD prevention manual guide was eventually created by the researchers with help from the Limpopo Department of Health for use by the clinics in the province of Limpopo. In addition, we helped capacitate participating clinics by making blood pressure machines, weighing scales, and measuring tapes available. In order to help with patient screening even after the initiative was over, participating CHWs were provided with a blood pressure monitor, an umbrella, and a squeeze bottle for carrying water. These steps were taken to support the expansion of community CVD screening, ongoing clinic referrals, health promotion, public awareness, and the long-term viability of this initiative for the prevention and management of CVDs (see Figure 2).

### 2.5. Data Collection

Community health workers (CHWs) used the non-laboratory INTERHEART Risk Score instrument to screen community members aged 18 and above who were found in their homes on the days that data were collected. Data were collected for a month in July 2021 by trained CHWs attached to the five clinics in the Ga-Molepo rural areas. This was followed by the rolling out of interventions from August 2021, a three-month follow-up, and another round of profiling in February 2022 after another three months. Data were collected and handled using the REDCap electronic data capture tool [28], which is hosted at the University of Antwerp and Makerere University.

### 2.6. The INTERHEART Risk Score Tool 

The INTERHEART Risk Score Tool that was utilized asked specific questions and each item was allocated a score as follows: being male ≥ 55 years and female ≥ 65 years = 2; being a smoker or having stopped 12 months ago or less = 2; smoking 1–5 cigarettes = 2; 6–10 = 4; 11–15 cigarettes = 6; 16–20 and 20 = 11; inhaling smoke from other people in the last 12 months = 2; indirect smoking last 12 months = 2; having diabetes = 6 and high blood pressure = 5; a parental history of heart attack = 4; stress and depression = 3 each; diet: consumption of salty food or snacks, fried food, no vegetables and fruits each scored 1; eating chicken or red meat twice a day = 2; inactive or performing mild physical exercise = 2; and measurement of waist-to-hip ratio (WHR) ≥ 0.874–0.963 = 2 and ≥0.964 = 4; [29]. The scores were added and those who scored 0–9 were deemed to be at a low risk, 10–15 were at a moderate risk, and 16–48 were at a high risk. The English version of the non-laboratory INTERHEART Risk Score Tool was translated by a language expert to Northern Sotho after conducting the pilot study. Northern Sotho is an indigenous language spoken by the majority of respondents in the Capricorn district of the Limpopo Province [26]. The tool was chosen because it can be used in settings with limited resources [29].

### 2.7. Measurements

The waist-to-height ratio circumference was measured using a tape measure. The waist circumference was measured at the approximate midpoint between the lower margin of the last palpable rib and the top of the iliac crest and the hip circumference was measured around the widest portion of the buttocks [30]. The blood pressure (BP) of those who didn’t know if they were hypertensive or not, was measured on the left arm using the MEDIC Pharmacists Choice Devices Blood Pressure Monitor Classic (MPCDBPMC). This BP Monitor has been clinically validated according to the European Society of Hypertension (ESH) protocol [31]. Being hypertensive included those who answered yes when asked if they had high blood pressure. Those who did not know that they were hypertensive, but were found to have a systolic BP score of ≥140 mmHg and diastolic BP score of ≥90 mmHg were regarded as being hypertensive [32].

### 2.8. Intervention Basket

The intervention entailed: Physical activities: daily brisk walking/jogging/riding a bicycle for 30 min or more than 5 days a week, and also being encouraged to start walking with friends for motivation and sustainability; Diet: eating a variety (at least 1) of vegetables and fruits daily, those with elevated waist/hip ratio were advised to reduce the food portions, reduction of salt in food at least 1 teaspoon a day, advised to check the amount of salt on the nutritional information provided on the package of readymade food, avoid eating junk food, reduction of red meat intake to 3 times a week, replacing meat with other proteins such as beans, masotsa, soya mince, lentils, boiled eggs, low fat milk, cutting fat from meat before cooking it; Smoking: those who were smoking were motivated to stop by applying the self-management strategy, advised to avoid indirect inhalation of cigarette smoke by not sitting next to people who are smoking, family members were not to smoke in the house or in the car; Reduction of stress: advised to avoid stress where possible, and if stress could not be avoided, they were educated about healthy ways of dealing with stress such as talking to a friend and creating time to relax, and those who could not cope were referred to the social worker or psychologist depending on the cause of the stress; Medication: To take medication for chronic diseases at the correct time and correct dose and to avoid taking unprescribed medication. Health advice pamphlets were distributed to the clients and their family members. 

### 2.9. Validity and Reliability

To ensure validity and reliability, the CHWs were first trained by the research team on risk factors associated with CVDs, prevention of CVDs and how to screen the participants using the INTERHEART Risk Score Tool for a week, followed by another week of training on intervention. They were also provided with 2 manuals for each training session. Three days of training for the CHWs were provided at their respective clinics and were continued before they started with the implementation. The INTERHEART Risk Score Tool has been validated by 52 countries [30]. The CHWs were supervised by registered nurses and the research team during data collection. 

### 2.10. Statistical Analysis

The difference between pre-intervention and post-intervention results was analyzed using a *t*-test and ANCOVA with age, smoking, hypertension, and diabetes as the covariates. To make potential contrasts between group means, the Scheffe post-hoc and Leven’s tests were used. The continuous variables and the results of ANCOVA were presented as means and confidence intervals. The categorical variables were expressed as numbers, charts, percentages, and confidence intervals. SPSS version 26.0 (IBM SPSS Statistics, Armonk, NY, USA) was used to perform the descriptive analyses (frequency, percentages, and cross-tabulation) and all the statistical tests. Statistical significance was accepted at *p* < 0.05.

## 3. Results

**Figure 3:**
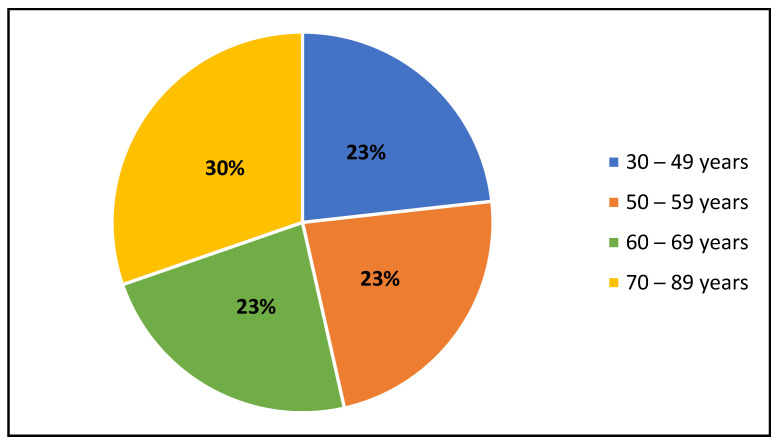
Age distribution of the community cohort study.

In this study, the researchers used an INTERHEART Risk Score Tool, involving 61 participants from the Ga Molepo rural villages, Limpopo Province, South Africa. Of the 61 participants, the majority were aged 60 and above (53%) followed by 50–59 years (23%). The majority of participants were females 47 (77%), as shown in Figure 3. The average age in years was 60 with a 95% confidence interval between 56.5 and 64.2 years. Ten percent of the participants smoked during the profiling phase, but none of them did so following the intervention. The results show that there was a decline post-intervention in terms of the high WHR [≥0.964] (from 20% to 9%), high salty and fried food consumption (from 65% to 16% and 18% to 9%, respectively), red meat/poultry consumption ≥ 2 times/day (from 45% to 2%), and mild exercise (from 55% to 22%), as shown in Table 1.

The results showed a significant difference in proportions pre- and post-intervention for raised SBP, obesity by BMI, and WC, only, at a 5% significant level, as shown in Table 2. 

The covariate greatly reduced the CI for the systole (mmHg) means, however for the other variables the mean slightly increased. The adjusted means obtained from the repeated ANCOVA results showed that the mean differences were still not statistically significant even when the covariates were included, as shown in Table 3.

**Figure 4:**
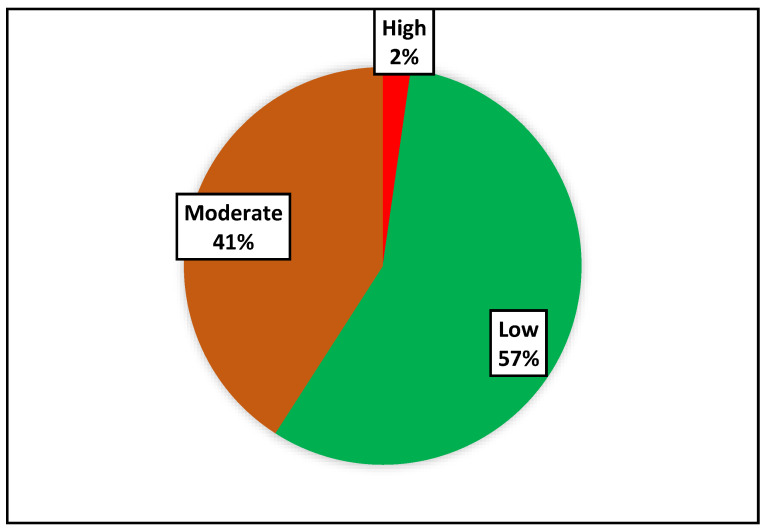
The risk level of developing a CVD among the community cohort study.

Of the 61 (100%) participants who were at a moderate risk of developing a CVD at the profiling stage, 57% moved to a low risk after the intervention whilst only 2% moved to a high risk, as shown in Figure 4.

## 4. Discussion

The purpose of this study was to assess the CVD risk in rural areas using the non-laboratory INTER-HEART Risk Score method and to implement comprehensive CVD preventive interventions utilizing self-management measures for individuals who were at a moderate risk. The CHWs received training on how to screen and implement these measures as well as CVD manuals on how to conduct risk assessments, health screenings, and educate people about CVDs. A study by Olmen and colleagues distilled three major issues for the management of chronic diseases’ rapid scale-up approaches through the public health approach, community and peer support, and the strengthening of the health services in which care is embedded [27]. The study followed these models to implement a comprehensive package for the prevention of CVDs. For scale-up approaches through the public health approach, through the study funding, we purchased blood pressure machines, glucometers, and scales for each participating clinic. We developed a CVD prevention manual guide to be used by the clinics in the Limpopo Province. In the community, we trained CHWs to conduct health education, risk assessments, motivational interviewing, and promote CVD health and education. We also bought each one of the CHWs measuring tapes, a blood pressure machine, an umbrella, and a squeeze bottle for carrying water to assist in screening patients even beyond the project.

A longitudinal, prospective cohort study by Lindbohm et al. (2021) assessed the risk of CVDs in primary care among participants who underwent clinical examinations at intervals of five years between 7 August 1991 and 6 December 2016, and were monitored for the occurrence of CVD until 2 October 2019 [33]. At the conclusion of the trial, it was discovered that participants at a high risk had raised their risk factor from 21% to 85% of developing a CVD, while participants at a moderate risk had decreased their risk factor from 15% to 8% over time [33]. Participants spontaneously migrated toward the high-risk level over time without any intervention. At the beginning of our study, 35% of the profiled individuals were at a moderate risk of developing a CVD, while 36% were at a high risk. Those who were at a high risk by the time of screening were referred to the clinics by the CHWs. Applying lifestyle changes, medical and psychosocial interventions, and health promotion and self-management strategies, are recommended by some prior researchers [22,23,28]. In this study’s post interventions, fewer moderate-risk participants consumed high-salt and fried foods, red meat/poultry ≥ 2 times/day, and performed mild exercise. We also had no smokers among our participants. It was pointed out by previous researchers that reducing the risk of CVDs can be achieved through regular exercise, quitting smoking, and achieving and maintaining a healthy diet [19].

The prevalence of moderate-risk participants in the study who had elevated SBP and were obese decreased significantly after the interventions. Achieving and maintaining a healthy weight, and controlling blood pressure, cholesterol, and blood sugar levels all helped to lower the risk of developing CVDs [19]. After following the intervention, only 18 (41%) were still at a moderate risk whilst 26 (57%) moved to a low risk. Unexpectedly, obesity was significantly elevated after the interventions. The COVID-19 alert levels that were in effect during the COVID-19 pandemic may have had an impact on this unanticipated outcome. Some of the participants may have spent some of their time bingeing on unhealthy foods and/or being sedentary because they had to work from home or were unable to exercise in public places, prevented from walking the streets or engaging in any form of physical activity in parks, stadiums, or gyms, which made the interventions difficult.

Despite several strengths of the study, including being an intervention study, the limitations should be noted. The study was a descriptive cohort study with a small sample and there was no control group. This means that every participant at a moderate risk received the intervention. The justification for the small sample size was that the study took place during the COVID-19 pandemic, and like everywhere else in the world, South Africa had put in place COVID-19 lockdown alert levels. As a result, some CHWs didn’t want to participate due to their worry and concern about the COVID-19 sickness spreading. Despite the difficulties and limitations, several CHWs and participants persisted until the conclusion of the intervention. Self-reported answers may be exaggerated or respondents may be too embarrassed to reveal private details or have forgotten some personal information and this can contribute to bias in the results. Further research is needed to assess the effect of the interventions by having case-control groups and also increasing the sample size.

## 5. Conclusions

This project increased the CHWs’ knowledge and skills to engage in CVD prevention and also the screening of the community for the risk of developing the disease using a non-laboratory risk score tool. Applying lifestyle modifications, medical and psychosocial interventions, as well as health promotion and self-management strategies, it was discovered that moderate-risk participants consumed less high-salt and fried foods, red meat/poultry two times per day, and engaged in light exercise after the interventions. Additionally, none of our participants were smokers after the interventions. The role of community health workers should be strengthened in its capacity to empower the community through health promotion strategies which address modifiable risk behaviors among the people in their households and communities. Efforts are needed in rural regions to promote CVDs and self-management strategies through educational programs such as encouraging patients to engage in increased physical activity and eating healthily. This basket of interventions will assist community members in reducing the risk of developing either metabolic syndromes or CVDs.

## Figures and Tables

**Table 1:** Repeated measurement of patients at a moderate risk of CVD.

Variables	July-2021 (*N* = 61) % (95% CI)	Aug-2021 (*N* = 52) % (95% CI)	Nov-2021 (*N* = 51) % (95% CI)	Feb-2022 (*N* = 46) % (95% CI)
Mean-Age	60.35 (56.5; 64.2)			
Smoking status				
Yes	10 (5; 21)	8 (3; 19)	2 (0.22; 12)	0
No	90 (79; 96)	92 (81; 97)	98 (88; 99)	100
Waist-to-hip ratio (WHR)				
<0.873	30 (20; 43)	36 (24; 50)	49 (36; 62)	37 (24; 53)
Between 0.873–0.963	50 (37; 63)	49 (36; 63)	42 (30; 56)	54 (38; 68)
≥0.964	20 (12; 32)	15 (7; 28)	9 (3; 19)	9 (3; 23)
Depressed/Stressed	47 (34; 60)			42 (28; 58)
High salty food consumption (≥1 time/day)	65 (52; 76)	43 (29; 58)	25 (15; 39)	16 (7; 31)
High-fried food/trans saturated fat consumption (≥3 times/week)	18 (10; 31)	20 (11; 35)	13 (6; 26)	9 (3; 23)
Low fruit consumption (<1 time/day)	48 (36; 61)	67 (53; 79)	44 (31; 58)	35 (21; 51)
Low Vegetable consumption (<1 time/day)	23 (14; 36)	31 (19; 45)	29 (18; 43)	14 (6; 28)
Red meat/poultry consumption ≥ 2 times/day	45 (33; 58)	18 (10; 32)	17 (9; 31)	2 (0.3; 16)
Mild exercise	55 (42; 67)	53 (39; 67)	53 (39; 67)	22 (13; 39)

**Table 2:** Pre-intervention against post-intervention results for patients at a moderate risk of CVD.

	Pre-Intervention		Post-Intervention		*p*-Value
**Variables**	**Mean (95% CI)**	**Min; Max**	**Mean (95% CI)**	**Min; Max**	
Body weight (kg)	74.75 (70.03; 79.46)	34; 118	76.74 (71.40; 82.09)	38; 117	0.285
Body height (m)	1.59 (1.57; 1.61)	1.39; 1.79	1.56 (1.51; 1.61)	0.58; 1.77	0.075
BMI (kg/m^2^)	29.47 (27.64; 31.30)	14.33; 48.13	30.43 (28.45; 32.42)	16.23; 43.50	0.075
Waist circumference (cm)	98.68 (95.02; 102.35)	75; 150	100.29 (96.54; 104.04)	70; 126	0.284
Waist-to-hip ratio (cm)	0.92 (0.87; 0.95)	0.71; 1.41	0.90 (0.87; 0.92)	0.76; 1.11	0.615
Systole	133.61 (127.65; 139.57)	85; 202	130.70 (124.70; 136.69)	86; 172.67	0.264
Diastole	79.69 (76.31; 83.08)	54.67; 116	81.46 (78.22; 84.69)	58.33; 102.33	0.268
**Prevalence**	**% (95% CI)**		**% (95% CI)**		
Obesity; BMI ≥ 30 kg/m^2^	50 (37; 63)		59 (44; 73)		<0.001 *
Obesity; WC ≥ 90 cm	78 (66; 87)		84 (70; 92)		<0.001 *
Obesity; WHR > 0.90	50 (37; 63)		52 (37; 67)		0.300
Raised SBP (mmHg)	37 (25; 50)		30 (18; 45)		0.006 *
Raised DBP (mmHg)	20 (12; 32)		25 (14; 40)		0.064

* Significant at 5% level.

**Table 3:** Repeated ANCOVA for pre-intervention against post-intervention results.

		Post-Intervention	
Variable	Pre-Intervention	Age-Adjusted	Age, Smoke, Hypertension, Diabetes-Adjusted
	Mean (95% CI)	Mean (95% CI)	Mean (95% CI)
Body weight (kg)	74.75 (70.03; 79.46)	76.74 (72.88; 80.61)	74.36 (68.40; 80.33)
Body mass index (kg/m^2^)	29.47 (27.64; 31.30)	30.44 (29.08; 31.79)	29.27 (27.27; 31.28)
Waist circumference (cm)	98.68 (95.02; 102.35)	100.29 (97.67; 102.88)	99.76 (95.71;103.79)
Waist-to-hip ratio (cm)	0.92 (0.87; 0.95)	0.90 (0.88; 0.93)	0.93 (0.89; 0.96)
Systole (mmHg)	133.61 (127.65; 139.57)	130.70 (125.38; 136.02)	130. 89 (122.53; 139.26)
Diastole (mmHg)	79.69 (76.31; 83.08)	81.47 (78.59; 84.32)	82.94 (78.56; 87.32)

## Data Availability

The data presented in this study are available on request from the corresponding author. The data are not publicly available due to privacy or ethical restrictions.
